# Supplementary material for: Hyperoxidation of mitochondrial peroxiredoxin limits H2O2‐induced cell death in yeast
Source: EMBO J. 2019 Aug 7;38(18):e101552. doi: 10.15252/embj.2019101552 (PMC6745495; doi:10.15252/embj.2019101552)
Supplement: Supplementary file 1 — Appendix [file EMBJ-38-e101552-s001.pdf]

Appendix

Hyperoxidation of mitochondrial peroxiredoxin limits H_2O_2-induced cell death in yeast

Gaetano Calabrese[1], Esra Peker[1], Prince Saforo Amponsah[2,3], Michaela Nicole Hoehne[1], Trine Riemer[1], Marie Mai[3], Gerd Patrick Bienert[4], Marcel Deponte[5], Bruce Morgan[3,*] and Jan Riemer[1,*]

1. University of Cologne, Department for Chemistry, Institute for Biochemistry, Zuelpicher Str. 47a, 50674 Cologne, Germany

2. University of Kaiserslautern, Department for Biology, Cellular Biochemistry, Erwin-Schroedinger Str. 13, 67663 Kaiserslautern, Germany

3. University of the Saarland, Institute of Biochemistry, Campus B 2.2, D-66123 Saarbruecken, Germany

4. Leibniz-Institute of Plant Genetics and Crop Plant Research (IPK), Department of Physiology and Cell Biology, Corrensstrasse 3, 06466 Gatersleben, Germany

5. University of Kaiserslautern, Department of Chemistry/Biochemistry, 67663 Kaiserslautern, Germany

* Correspondence to JR, lead contact: Tel.: +49-221-470-7306, Email: jan.riemer@uni-koeln.de

* Correspondence to BM: Tel.: +49-681-302-3339, Email: bruce.morgan@uni-saarland.de

Table of Content - Appendix

Appendix Figures

Appendix Figure **S1**	Page 3
Appendix Figure **S2**	Page 4
Appendix Figure **S3**	Page 5
Appendix Figure **S4**	Page 6
Appendix Figure **S5**	Page 9
Appendix Figure **S6**	Page 10

Appendix Tables

Appendix Table S1. Yeast strains used in this study.	Page 12
Appendix Table S2. Plasmids used in this study	Page 13

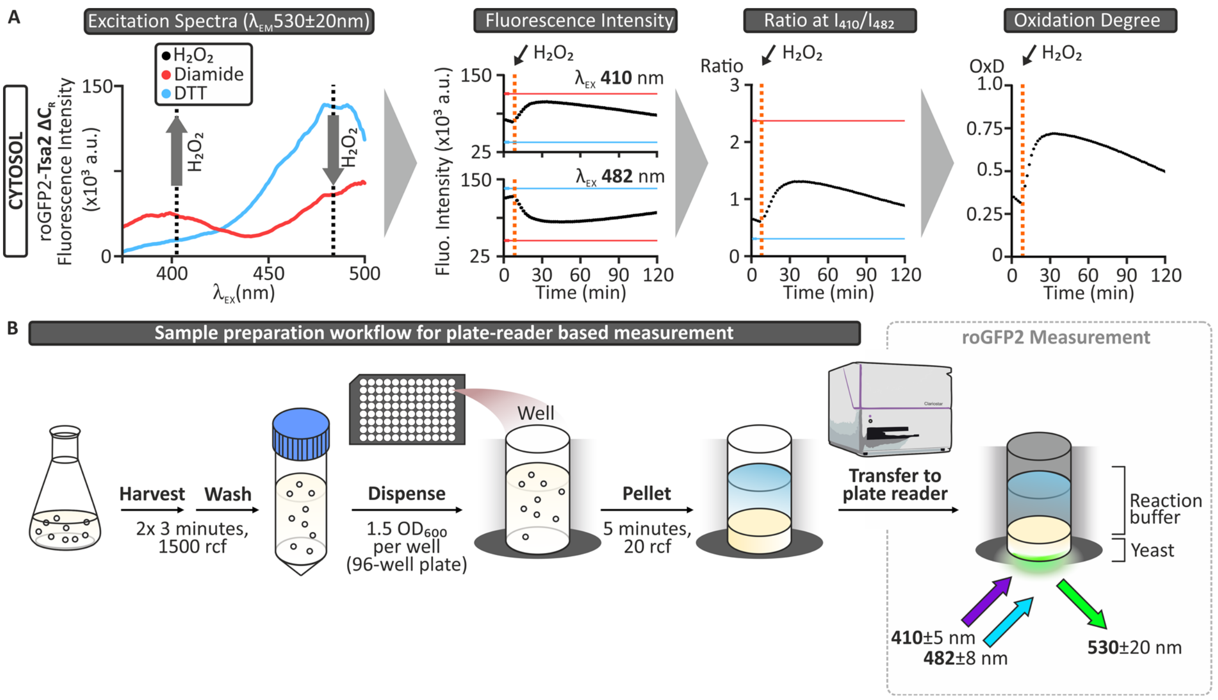

Appendix Figure S1. (corresponds to Figure 1)

(A) Explanation of the principles and necessary calculation steps to interpret the responses of roGFP2-based sensors to oxidative and reductive challenges Refer to the “*Fluorescence measurement of roGFP2-sensor oxidation*” section of Materials and Methods for a detailed explanation. RoGFP2-based sensors are ratiometric sensors. Upon oxidation or reduction, the excitation spectrum of roGFP2 changes so that at two excitation wavelengths (410, 482 nm) fluorescence emission intensity changes in opposite directions. From these data it is possible to calculate either a ratio of fluorescence emission intensity, which can be compared to the ratio of fully oxidized and fully reduced controls samples, or to calculate the degree or roGFP2 oxidation, based upon Equation 1, Material and Methods.

(B) Cartoon model representing the workflow to perform a “*Fluorescence measurement of roGFP2-sensor oxidation*”. Cells grown to exponential phase are harvested and washed, and resuspended to a density of 7.5 OD_{600} units/ml. From this cell suspension 180 µl (i.e. 1.5 OD_{600} units) are transferred to each well of a flat-bottomed 96-well plate. Yeast cells are pelleted to the bottom of the plate well via a mild centrifugation step. RoGFP2 dynamics are then measured in a fluorescence plate reader, where the fluorescence is recorded at λ_{EM} 530±20nm with excitation at λ_{EX} 410±5nm and λ_{EX} 482±8nm. After the 5th cycle, 20 µl of a 10x solution of the desired chemical to test is added gently to the reaction (final 1x).

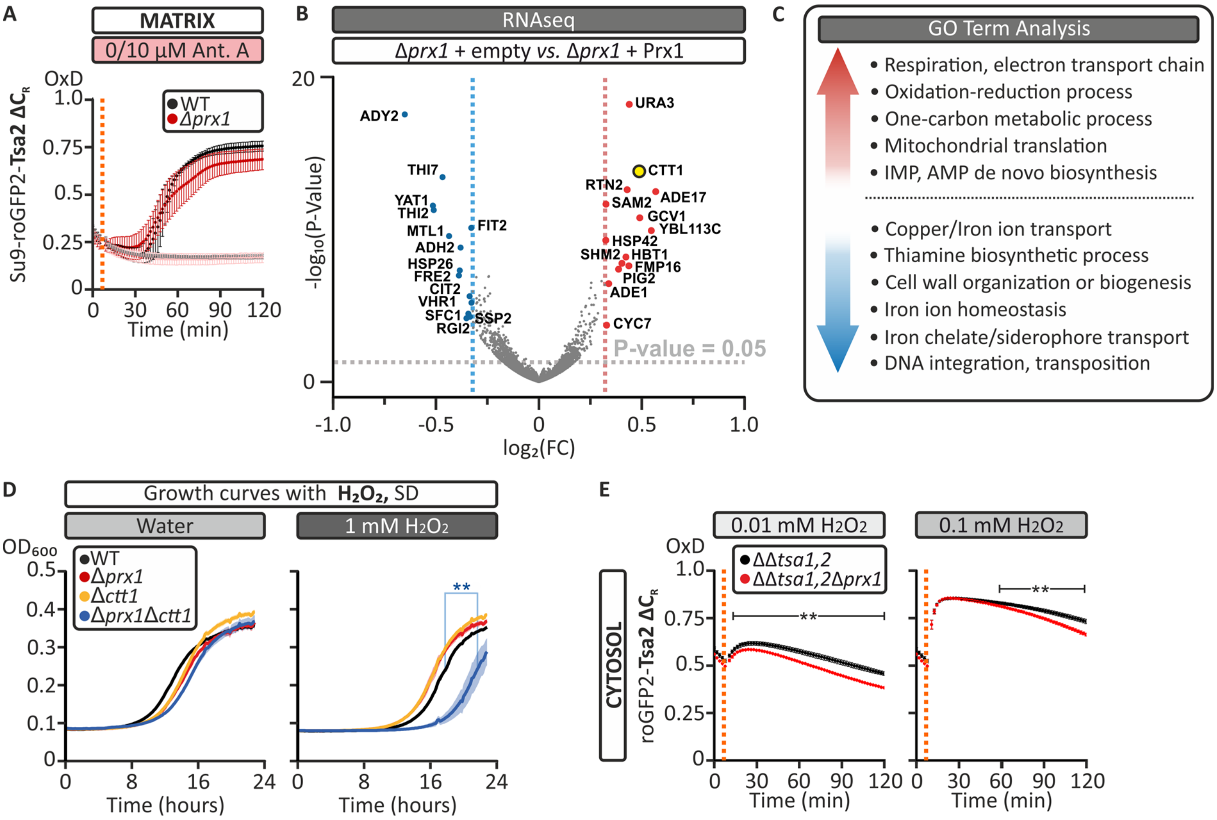

Appendix Figure S2. (corresponds to Figure 2)

(A) The response of mitochondrial matrix-localized roGFP2-Tsa2ΔC$_R$ probe, in wild-type and *Δprx1* cells to the addition of 10 µM antimycin A. Cells were grown in SGal (-Leu) medium and harvested at early exponential phase. Lighter colored curves are controls showing the probe response upon the addition of 0.1% (v/v) ethanol.

(B) Volcano plot of the differential mRNA expression in *Δprx1*+empty vector cells, compared to *Δprx1*+Prx1-WT cells. P-values are plotted against enrichment (log_2(fold change (FC))). Cut-off was set at ±0.32 log_2(fold change (FC)). Cells were grown in SGal (-Ura) medium and harvested at early exponential phase.

(C) The cellular process GO terms analysis of the differential expression output data represented in (B) performed using the online tool *GOrilla*.

(D) Growth curve of wild-type, *Δprx1, Δctt1* and *Δctt1Δprx1* cells in SD medium complemented with all amino acids and 1 mM H_2O_2 or water as a control. (n = 3 biological replicates with cells obtained from independent cultures for each replicate). Significance for the difference in the time the cultures reach 50% of their maximal OD_{600} was assessed with a Student's, 2-tailed, unpaired t-test. **p < 0.01. Compared to the other strains, *Δctt1Δprx1* cells have an extended lag phase.

(E) The response of cytosolic roGFP2-Tsa2ΔC$_R$ probe, in *Δtsa1Δtsa2* and *Δtsa1Δtsa2Δprx1* cells grown in SGal (-Leu) medium, to the addition of 0.1 mM exogenous H_2O_2. Both strains exhibit very similar responses with the *Δtsa1Δtsa2Δprx1* cells allowing for slightly faster recovery of the cytosolic roGFP2-Tsa2ΔC$_R$ probe after bolus H_2O_2 incubation.

OxD refers to the degree of sensor oxidation. Error bars represent the standard deviation (n = 3 biological replicates, with cells taken from independent cultures for each individual biological replicate). Significance was assessed with the t-test. **p < 0.01.

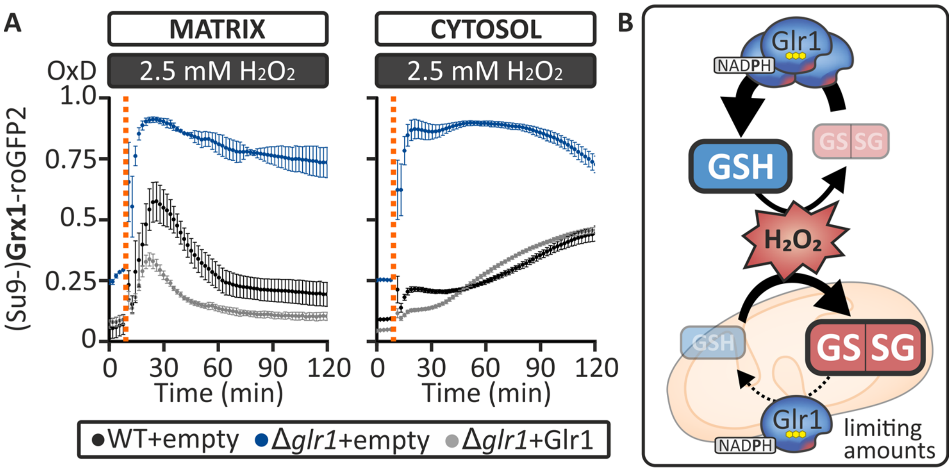

Appendix FigureS3. (corresponds to Figure 3)

(A) The response of cytosolic and mitochondrial matrix-localized Grx1-roGFP2 probes, expressed in BY4742 wild-type yeast cells with an empty vector or in *Δglr1* cells transformed either with an empty vector or a vector encoding wild-type Glr1 grown in SGal (-Leu,-Ura), to a bolus of exogenous 2.5 mM H_2O_2. Expression of Glr1 from a strong promotor improves maintenance of E_{GSH} in the matrix compared to wild-type cells indicating the presence of only limiting amounts of Glr1 in the matrix of wild-type cells.

(B) Cartoon model representing that Glr1 activity in the matrix is limiting for reduction of glutathione disulfide.

OxD refers to the degree of sensor oxidation. Error bars represent the standard deviation (n = 3 biological replicates, the mean of 3 technical replicates in each case, in which the probe response was measured 3 times, with cells obtained from 3 independent cultures, for every strain and probe combination).

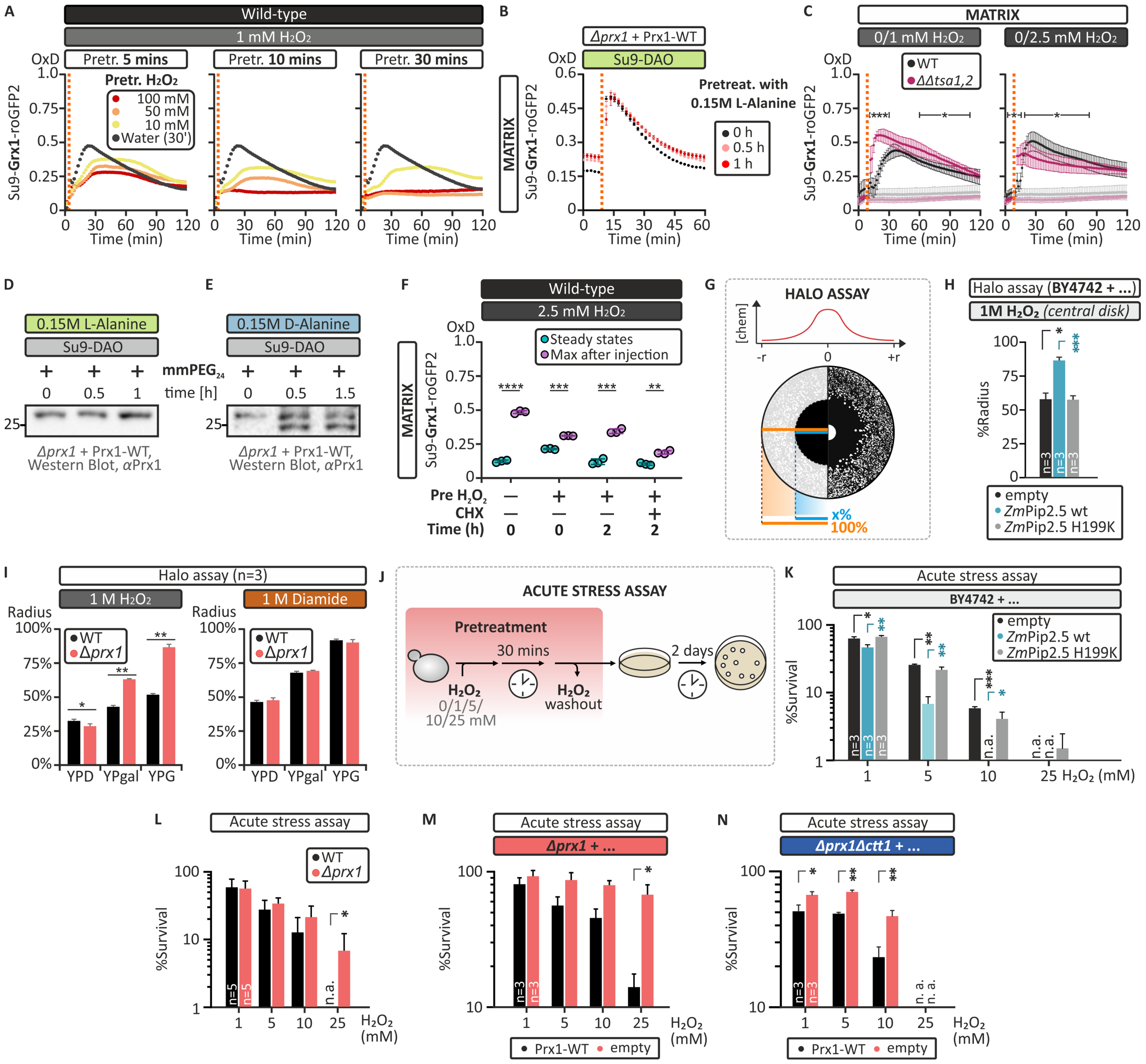

Appendix Figure S4. (corresponds to Figure 4)

(A) '*Acute stress-washout*' assay. BY4742 wild-type yeast cells expressing a mitochondrial matrix-localized Grx1-roGFP2 probe were pre-treated for the indicated times with the indicated amounts of H_2O_2. Afterwards, cells were washed and the response of matrix-targeted Grx1-roGFP2 to the addition of 1 mM H_2O_2 was measured. For this experiment cells were grown in SGal (-Leu) medium and harvested at early exponential phase. Preincubation with H_2O_2 attenuates the subsequent response of the Grx1-roGFP2 probe towards a 1 mM bolus application of H_2O_2.

(B) Δ*prx1* cells transformed with a plasmid encoding wild-type Prx1, a plasmid encoding the matrix-targeted D-amino acid oxidase (Su9-DAO) and expressing a mitochondrial matrix-localized Grx1-roGFP2 were grown in SGal (-Leu -Ura -His) to early exponential phase before the addition of 0.15 M ***L***-alanine for the indicated times. Subsequently, cells were washed and the response of Grx1-roGFP2 to the addition of 1 mM H_2O_2 was measured. Incubation with L-alanine (not a substrate of DAO) does not affect the Grx1-roGFP2 response.

(C) The response of mitochondrial matrix Grx1-roGFP2, expressed in wild-type and Δ*tsa1*Δ*tsa2* cells, to the addition of exogenous H_2O_2. Cells were grown in in SGal (-Leu). Δ*tsa1*Δ*tsa2* cells exhibit at higher H_2O_2 concentrations a lower response of the matrix-targeted Grx1-roGFP2

probe. In these cells higher amounts of H_2O_2 can reach the matrix and we propose can there hyperoxidize Prx1 impairing oxidation of GSH during bolus H_2O_2 treatment. Error bars represent the standard deviation (n = 3 biological replicates, the mean of 3 technical replicates in each case, in which the probe response was measured 3 times, with cells obtained from 3 independent cultures).

(D) Redox shift assay to establish the redox state of Prx1 in the samples of (**B**). The experiment was performed as described in **Figure 4F**. The redox state of Prx1 does not change upon addition of ***L***-alanine

(E) Redox shift assay to establish the redox state of Prx1 in the samples of **Figure 4D**. The cysteine C91 in Prx1 becomes partially inaccessible upon incubation of DAO-expressing cells with ***D***-alanine. This is in line with the decreased Grx1-roGFP2 response in **Figure 4D** and argues for hyperoxidation of Prx1 under these conditions.

(F) The graph shows steady state Grx1-roGFP2 oxidation following H_2O_2 pre-treatment and cytosolic translation inhibition as well as the maximum probe oxidation in response to the subsequent second H_2O_2 treatment. Data refers to the experiment performed in **Figure 4J**.

(G) Graphic representation of the 'Halo assay' for growth sensitivity upon chronic exposure to H_2O_2. The zone of growth inhibition is reported as the percentage of the radius of the circle where no colonies grow in relation to the maximal radius of the plate. The example plate is representative for one of the three biological replicates of the experiment depicted in (**H**), specific to BY4742 + empty plasmid.

(H) H_2O_2 sensitivity of yeast cells expressing *Zea mays* aquaporins in their plasma membrane. Cultures of wild-type *S. cerevisiae* cells (BY4742) co-transformed with empty plasmid, or an plasmid encoding active aquaporin PIP25 or the inactive H199K mutant, were diluted in sterile distilled water to an OD_{600} of 0.1 and dispersed on plates SD (-Leu) agar plates. A filter disk placed in the middle of the plate was infused with 1 M H_2O_2 and the zone of growth inhibition "Halo" was recorded after 2 days growth at 30°C. Cells expressing a functional aquaporin in the plasma membrane are more sensitive to chronic H_2O_2 stress. (n = 3 biological replicates, with cells taken from independent cultures for each individual biological replicate).

(I) H_2O_2 and diamide sensitivity of yeast cells lacking the mitochondrial peroxiredoxin Prx1. Cultures of wild-type and Δ*prx1* cells were diluted in sterile distilled water to an OD_{600}/mL of 0.1 and dispersed on plates containing the different carbon sources glucose (YPD, fermentation), galactose (YPGal, fermentation and respiration), and glycerol (YPG, respiration is enforced). A filter disk placed in the middle of the plate was infused with 1 M H_2O_2 or 1 M diamide and the zone of growth inhibition was recorded after 2 days growth at 30°C. (n = 3 biological replicates, with cells taken from independent cultures for each individual biological replicate).

(J) Layout of the 'acute stress assay' experiment preformed in (**K, L, M, N**) and in **Figures 5E, 6A, 7C-D** – refer to the "*Acute stress assay*" section of *materials and methods* for a detailed explanation**.**

(K) H_2O_2 'acute stress' assay for yeast cells expressing *Zea mays* aquaporins in their plasma membrane. Cultures of wild-type *S. cerevisiae* cells (BY4742) co-transformed with an empty plasmid or a plasmid encoding, active aquaporin PIP25 or the inactive H199K mutant were grown in SD (-Leu) medium and harvested at early exponential phase. Cells were pre-treated with the indicated amounts of H_2O_2 for 30 mins. Afterwards, the cells were diluted and a fixed volume plated on YPD plates. The number of viable colonies was counted after 2 days growth at 30°C, here represented as a percentage with respect to the 0 mM pre-treatment. Cells expressing a functional aquaporin in the plasma membrane are more sensitive to acute H_2O_2

stress. Error bars represent standard deviation (n = 3 biological replicates, with cells taken from independent cultures for each individual biological replicate).

(L) H_2O_2 'acute stress' assay. Wild-type and Δ*prx1* cells pre-grown in YPD to early exponential phase were treated with the indicated amounts of H_2O_2 for 30 mins. Afterwards, the cells were diluted and a fixed volume plated on YPD plates. The number of viable colonies was counted after 2 days growth at 30°C, here represented as a percentage in respect of the 0 mM pre-treatment. Cells lacking Prx1 are more resistant to acute H_2O_2 treatment. Error bars represent standard deviation (n = 5 biological replicates, with cells taken from independent cultures for each individual biological replicate).

(M) H_2O_2 'acute stress' assay. Δ*prx1* cells co-transformed with either an empty vector or a plasmid encoding wild-type Prx1 were grown in SGal (-Ura) to early exponential phase. Cells were subsequently treated with the indicated amounts of H_2O_2 for 30 mins. Afterwards, the cells were diluted and a fixed volume plated on YPD plates. The number of viable colonies was counted after 2 days grown at 30°C, here represented as a percentage in respect of the 0 mM pre-treatment. Δ*prx1* cells with reintroduced Prx1 are more sensitive to acute H_2O_2 stress. Error bars represent standard deviation (n = 3 biological replicates, with cells taken from independent cultures for each individual biological replicate).

(N) H_2O_2 'acute stress' assay. Δ*prx1*Δ*ctt1* cells co-transformed with an empty plasmid or with a plasmid encoding wild-type Prx1 were grown in SGal (-Ura) and harvested at early exponential phase. Cells were subsequently treated with the indicated amounts of H_2O_2 for 30 mins. Afterwards, the cells were diluted and a fixed volume plated on YPD plates. The number of viable colonies was counted after 2 days grown at 30°C, here represented as a percentage in respect of the 0 mM pre-treatment. Cells lacking Prx1 are more resistant to acute H_2O_2 stress even in the absence of the *CTT1* gene. This indicates that a main contribution to acute H_2O_2 resistance stems from properties of Prx1 and not the upregulation of Ctt1 during an adaptive response. Error bars represent standard deviation (n = 3 biological replicates, with cells taken from independent cultures for each individual biological replicate).

In all graphs, significance was assessed with a Student's, 2-tailed, unpaired t-test. *$p < 0.05$; **$p < 0.01$; ***$p<0.001$;****$p < 0.0001$.

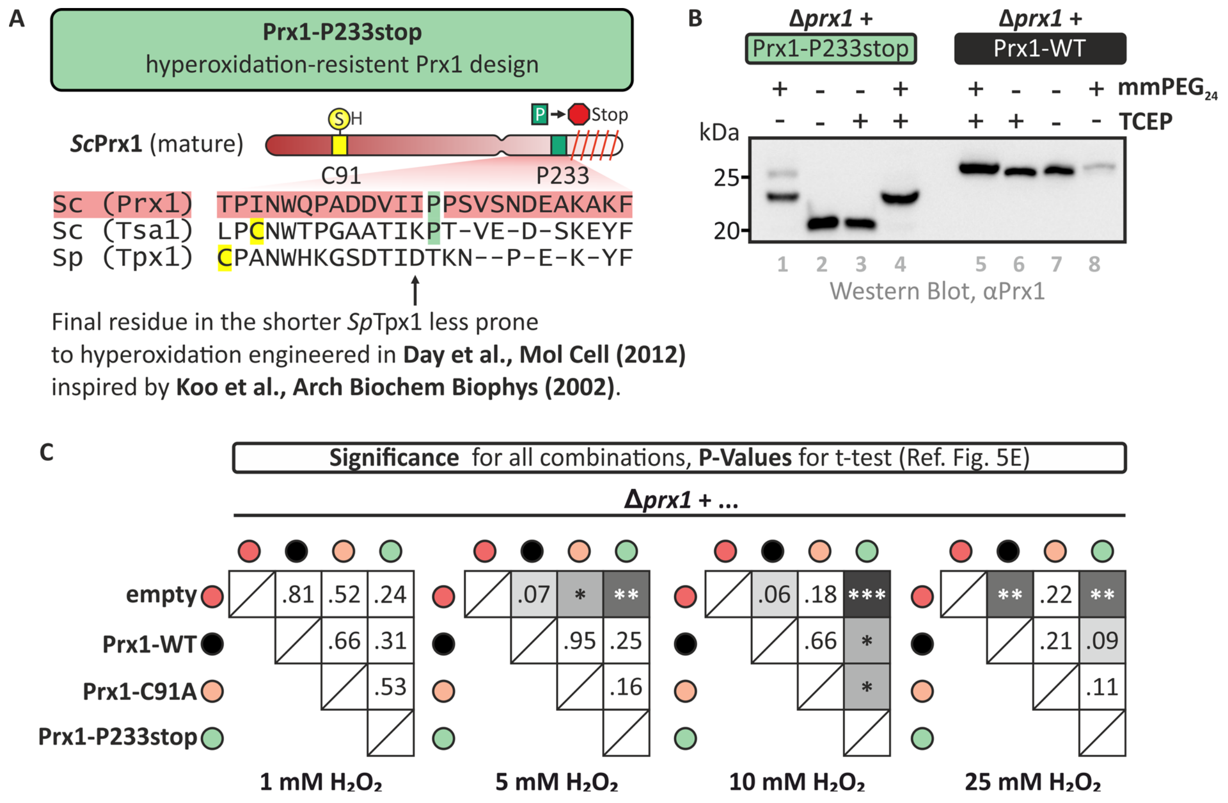

Appendix Figure S5. (corresponds to Figure 5)

(A) Scheme illustrating the rationale behind the design of Prx1-P233stop. Alignment of amino acids in the C-terminal region of *S. cerevisiae* Prx1, *S. cerevisiae* Tsa1 and *S. pombe* Tpx1. A truncated *Sp*Tpx1 variant (truncation site indicated by the arrow) is more resistant to hyperoxidation by H_2O_2. We tested whether truncation of Prx1 at an analogous position would render Prx1 more resistant to H_2O_2-induced hyperoxidation. Collectively, the data in **Figures 5** and **6** indicate that this is indeed the case. At present, the molecular basis of this increased resistance remains unclear.

(B) Redox shift assays of Prx1 variants in Δ*prx1* transformed with a plasmid encoding the Prx1-P233stop variant or wild-type Prx1 to assess Prx1-P233stop migration behavior on SDS-PAGE and assess accessibility to mmPEG in shift experiments. Prx1-P233stop can be shifted by mmPEG and at steady state (lane 1) is fully reduced. At the resolution of this gel, differences in full-length Prx1 migration behavior cannot be deduced.

(C) Tables summarizing the significance assessed with a Student's, 2-tailed, unpaired t-test for all the combination of the strains used in **Figure 5E**. *p < 0.05; **p < 0.01; ***p < 0.001;****p < 0.0001.

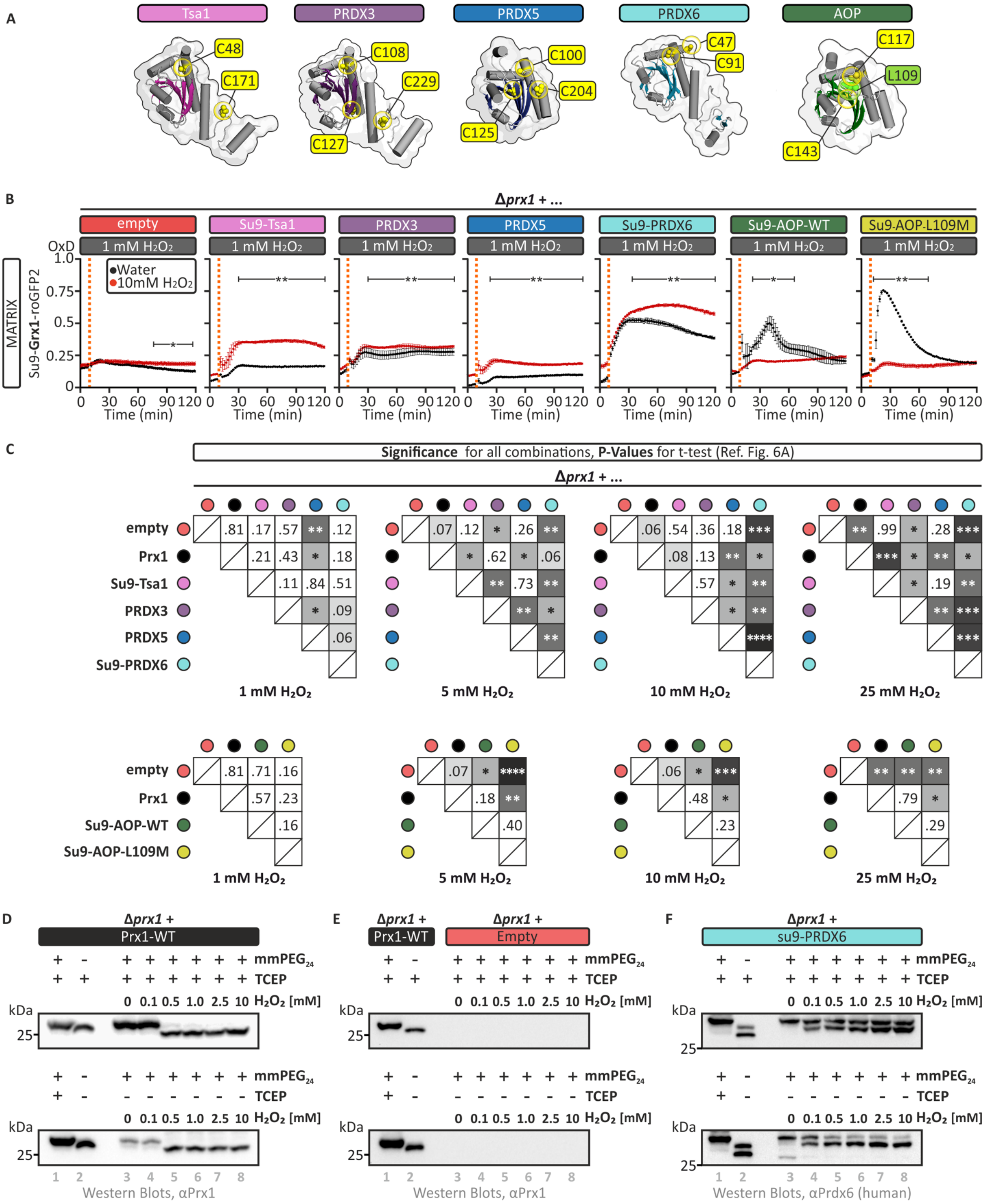

Appendix Figure S6. (corresponds to Figure 6)

(A) Structure of Tsa1 (PDB ID: 3SBC), PRDX3 (PDB ID: 5UCX), PRDX5 (PDB ID: 1HD2), PRDX6 (PDB ID: 1PRX), *Pf*AOP (PDB ID: 1XIY). Positions of relevant amino acids are indicated.

(B) The response of a mitochondrial matrix-localized Grx1-roGFP2 probe, expressed in Δ*prx1* cells containing an empty plasmid or a plasmid encoding either Su9-sa1, PRDX3, PRDX5 or Su9-PRDX6, Su9-*Pf*AOP or Su9-*Pf*AOP-L109M, to the addition of 1 mM exogenous H_2O_2 after a preceding pretreatment with either 10 mM H_2O_2 or water as a control (acute stress assay). Some peroxiredoxins mediate efficient GSH oxidation (*e.g.* HsPRDX6, AOP L109M), others are less so (*e.g.* HsPRDX5). After preincubation with H_2O_2, transfer of oxidation from H_2O_2 onto

E_{GSH} is prevented for some peroxiredoxins (*e.g.* AOP L109M), for others not (*e.g.* HsPRDX6). Error bars represent the standard deviation (n = 3 biological replicates, the mean of 3 technical replicates in each case, in which the probe response was measured 3 times, with cells obtained from 3 independent cultures, for every strain). OxD represents the degree of sensor oxidation. Cells were grown to an early exponential phase in SGal medium lacking the appropriate amino acids for plasmid selection.

(C) Tables summarizing the significance assessed with a Student's, 2-tailed, unpaired t-test for all the combination of the strains used in **Figure 6A**. *p < 0.05; **p < 0.01; ***p < 0.001;****p < 0.0001.

(D) Effect of acute H_2O_2 exposure on Prx1 redox state. Δ*prx1* cells co-transformed with a plasmid encoding wild-type Prx1 and grown in SGal (-Ura) medium to early exponential phase were treated with the indicated amounts of H_2O_2 for 10 mins. Afterwards, the cells were either directly modified with the alkylating agent $mmPEG_{24}$ or first reduced with TCEP (reduction of disulfide bonds and sulfenylated cysteines, but not hyperoxidized sulfinylated and sulfonylated cysteines) and then modified with $mmPEG_{24}$. The lower band present in the TCEP-treated samples indicates and irreversible hyperoxidation of the cysteine. At around 0.5 mM exogenous H_2O_2 (in some assays at 1 mM) Prx1 becomes hyperoxidized (observe TCEP treatment does not reduce the blocked cysteine residue).

(E) Effect of acute H_2O_2 exposure on cells lacking *PRX1*. Δ*prx1* cells co-transformed with an empty plasmid and grown in SGal (-Ura) medium to early exponential phase were treated with the indicated amounts of H_2O_2 for 10 mins. Afterwards, the cells were either directly modified with the alkylating agent $mmPEG_{24}$ or first reduced with TCEP and then modified with $mmPEG_{24}$. The controls for Δ*prx1* cells expressing wild-typePrx1 from a plasmid are provided. In Δ*prx1* cells, no band can be observed indicating specificity of the antibody.

(F) Effect of acute H_2O_2 exposure on Δ*prx1* cells complemented with a plasmid encoding Su9-PRDX6. Cells were grown in SGal (-His) and treated with the indicated amounts of H_2O_2 for 10 mins. Afterwards, the cells were either directly modified with the alkylating agent $mmPEG_{24}$ or first reduced with TCEP and then modified with $mmPEG_{24}$. At around 0.5 mM external H_2O_2 PRDX6 becomes inaccessible to mmPEG modification. This is however not hyperoxidation as it can be reverted by treatment with TCEP. The PRDX6 cysteine remains (at least partially) in a non-hyperoxidized state upon treatment with up to 10 mM H_2O_2.

Tables

Appendix Table S1. Yeast strains used in this study.

Strain	Genotype	Source
**BY4742**	*MATα his3Δ1 leu2Δ1 lys2Δ0 ura3Δ0*	Euroscarf
Δtsa1Δtsa2	BY4742 *Δtsa1::natNT2, Δtsa2::kanMX4*	Morgan et al. Nat Chem Bio (2016)
Δpor1	BY4742 *Δpor1::kanMX4*	Euroscarf
Δprx1	BY4742 *Δprx1::kanMX4*	Euroscarf
Δtrx3	BY4742 *Δtrx3::kanMX4*	Euroscarf
Δctt1	BY4742 *Δctt1::kanMX4*	Euroscarf
Δctt1Δprx1	BY4742 *Δprx1::kanMX4, Δctt1::HIS3MX6*	**This study**
Δtrr2	BY4742 *Δtrr2::kanMX4*	Euroscarf
Δtsa1Δtsa2Δprx1	BY4742 *Δtsa1::natNT2, Δtsa2::kanMX4, Δprx1::hphNT1*	**This study**
Δglr1	BY4742 *Δglr1::kanMX4*	Euroscarf
Δglr1Δprx1	BY4742 *Δglr1::kanMX4, Δprx1::hphNT1*	**This study**

Appendix Table S2. Plasmids used in this study

Plasmid name	Description	Marker	Primers (5' → 3')	Source
**p415 TEF Empty**	TEF promoter, CEN-plasmid	LEU2	\	Mumberg et al. (1995)
**p416 TEF Empty**	TEF promoter, CEN-plasmid	URA3	\	Mumberg et al. (1995)
**p415 TEF roGFP2-Tsa2ΔC_R**	roGFP2 fused to Tsa2ΔC_R [CYTOSOL]	LEU2	\	Morgan et al. Nat Chem Bio (2016)
**p415 TEF Su9-roGFP2-Tsa2ΔC_R**	roGFP2-Tsa2ΔC_R fused to presequence (1-69) of subunit 9 of Neurospora crassa ATPase [MATRIX]	LEU2	Su9 [XbaI-BamHI]<br>F: TCTAGAATGGCCTCCACTCGTG<br>R: GGATCCGGAAGAGTAGGCGCGC<br><br>roGFP2-Tsa2ΔC_R [BamHI-XhoI]<br>F:GGATCCACCGCTAGCGAATTTTCAAAGGG<br>R:CTCGAGTTAATTATTGGCATTTTTGAAATACTCC	**This Study**
**p415 TEF Grx1-roGFP2**	Grx1 fused to roGFP2 [CYTOSOL]	LEU2	\	Morgan et al. Nat Chem Bio (2011)
**p415 TEF Su9-Grx1-roGFP2**	Grx1-roGFP2 fused to presequence (1-69) of subunit 9 of Neurospora crassa ATPase [MATRIX]	LEU2	\	Kojer et al. MBOC (2015)
**p415 TEF Su9-roGFP2**	roGFP2 fused to presequence (1-69) of subunit 9 of Neurospora crassa ATPase [MATRIX]	LEU2	\	Kojer et al. MBOC (2015)
**p416 TEF Su9-Grx1-roGFP2**	Grx1-roGFP2 fused to presequence (1-69) of subunit 9 of Neurospora crassa ATPase [MATRIX]	URA3	\	Kojer et al. MBOC (2015)
**p415 TEF Su9-roGFP2-Prx1**	roGFP2-Prx1 fused to presequence (1-69) of subunit 9 of Neurospora crassa ATPase [MATRIX]	LEU2	Su9 [XbaI-BamHI]<br>F: TCTAGAATGGCCTCCACTCGTG<br>R:GGATCCGGAAGAGTAGGCGCGC<br><br>roGFP2-Prx1 [BamHI-XhoI]<br>F:GGATCCGTGAGCAAGGGCGAGGAGC<br>R:ctcgagTTAtttcgacttggtgaatcttaaataggg<br><br>Template: p416 roGFP2-Prx1 from Morgan et al. Nat Chem Bio (2016)	**This Study**
**p415 TEF Su9-roGFP2-Prx1-P233stop**	roGFP2-Prx1-P233stop fused to presequence (1-69) of subunit 9 of Neurospora crassa ATPase [MATRIX]	LEU2	Su9 [XbaI-BamHI]<br>F: TCTAGAATGGCCTCCACTCGTG<br>R:GGATCCGGAAGAGTAGGCGCGC<br><br>roGFP2-Prx1-P233stop [BamHI-XhoI]<br>F:GGATCCGTGAGCAAGGGCGAGGAGC<br>R: ctcgagTCAaataatgacatcgtcagctggctgcc<br><br>Template: p415 TEF su9-roGFP2-Prx1 from this study	**This Study**

**pEPT Prx1-WT**	*PRX1* with **E**ndogenous **P**romoter and **T**erminator (CDS ± flanking regions)	URA3	*(-600bp)_ PRX1_(+381bp)* [SacI-KpnI]<br>F:GAGCTCagacatatataaagaactaagaaaag<br>R:GGTACCgtgagatattcgttgtttcttac<br><br>Cloned in p416 TEF Empty	**This Study**
**pEPT Prx1-C91S**	Prx1 with C91S mutation (site directed mutagenesis of pEPT Prx1-WT)	URA3	Quick-change primers<br><br>Prx1_C91A_fw:<br>cagatttcacccctgtcGCCaccaccgaagtcagc<br>Prx1_C91A_rev:<br>gctgacttcggtggtGGCgacaggggtgaaatctg	**This Study**
**pEPT Prx1-P233stop**	Prx1 with P233stop mutation (site directed mutagenesis of pEPT Prx1-WT)	URA3	Quick-change primers<br><br>Prx1_P233stop_fw:<br>gccagctgacgatgtcattattTGAccctctgtctccaatgatg<br>Prx1_P233stop_rev:<br>catcattggagacagagggTCAaataatgacatcgtcagctggc	**This Study**
**p416 TEF Su9-AOP-WT**	*Pf*AOP fused to presequence (1-69) of subunit 9 of Neurospora crassa ATPase [MATRIX]	URA3	\	Staudacher et al. Redox Biology (2018)
**p416 TEF Su9-AOP-L109A**	Su9-*Pf*AOP with L109A mutation (site directed mutagenesis of p416 su9-PfAOP-WT) [MATRIX]	URA3	\	Staudacher et al. Redox Biology (2018)
**p416 TEF Su9-AOP-L109M**	Su9-*Pf*AOP with L109M mutation (site directed mutagenesis of p416 su9-PfAOP-WT) [MATRIX]	URA3	\	Staudacher et al. Redox Biology (2018)
**p416 TEF Su9-Tsa1-3HA**	Tsa1 fused to presequence (1-69) of subunit 9 of Neurospora crassa ATPase and with 3x Hemagglutinin tag [MATRIX]	URA3	Su9 [XbaI-BamHI] as above<br><br>Tsa1 [BamHI-XhoI]<br>F:GGATCCGTCGCTCAAGTTCAAAAGCAAGC<br>R:CTCGAGTTTGTTGGCAGCTTCGAAGTATTCC<br><br>3HA already in the plasmid, with final TAA	**This Study**
**p416 TEF PRDX3-3HA**	PRDX3 fused with 3x Hemagglutinin tag [MATRIX, has MTS]	URA3	PRDX3 [XbaI-SalI]<br>F: tctagaATGGCGGCTGCTGTAGG<br>R: gtcgacCTGATTTACCTTCTGAAAGTACTCTTTGG<br><br>3HA already in the plasmid, with final TAA	**This Study**
**p415 TEF PRDX5-His6**	PRDX5 fused with Hexahistidine tag [MATRIX, has MTS]	LEU2	PRDX5 [XbaI-SalI]<br>F:TCTAGAatgggactagctggcgtgtgcg<br>R:GTCGACgagctgtgagatgatattgggtgcc<br><br>His_6 already in the plasmid, with final TAA	**This Study**

**p413 TEF Su9-PRDX6-FLAG**	PRDX6 fused to presequence (1-69) of subunit 9 of Neurospora crassa ATPase and with FLAG tag [MATRIX]	HIS3	Su9 [XbaI-BamHI] as above<br><br>PRDX6 [BamHI-SalI]<br>F:AGATCTcccggaggtctgcttctcgggg<br>R:GTCGACaggctggggtgtgtagcggagg<br><br>FLAG already in the plasmid, with final TAA	**This Study**
**p416 TEF Glr1-WT-3HA**	Glr1 fused to presequence (1-69) of subunit 9 of Neurospora crassa ATPase and with 3x Hemagglutinin tag [CYTOSOL+MATRIX]	URA3	Glr1 [XbaI-SalI]<br>F:TCTAGAatgctttctgcaaccaaacaaacatttag<br>R:GTCGACtcatctcatagtaaccaattcttctgc<br><br>3HA already in the plasmid, with final TAA	**This Study**
**p416 TEF cyto-Glr1-3HA**	Glr1Δ1-16 with 3x Hemagglutinin tag [CYTOSOL]	URA3	Glr1 [XbaI-SalI]<br>F:TCTAGAatgCCCGGGtccacgaacaccaagcattacg<br>R:GTCGACtcatctcatagtaaccaattcttctgc<br><br>3HA already in the plasmid, with final TAA	**This Study**
**p416 TEF Trx3-3HA**	Trx3 with 3x Hemagglutinin tag [MATRIX]	URA3	Trx3 [XbaI-SalI]<br>F: TCTAGAatgttgttctataagcctgtgatgagg<br>R: GTCGACtagatctttgattcccttctctaaagc<br><br>3HA already in the plasmid, with final TAA	**This Study**
**p413 *GPD* su9-DAO-FLAG**	D-Aminooxidase fused to presequence (1-69) of subunit 9 of Neurospora crassa ATPase and with FLAG tag [MATRIX]	HIS3	Su9 [XbaI-BamHI] as above<br><br>DAO [BamHI-SalI]<br>F: GGATCCcacagccagaagagggtgg<br>R: GTCGACgctctccctagctgcgc<br>*Template source:* Matlashov et al. ARS (2014)<br><br>FLAG already in the plasmid, with final TAA	**This Study**
**pRS425 *pTPIu* ZmPIP25-WT**	Zea mais PIP2;5 aquaporin codon-optimized for yeast expression [PLASMA MEMBRANE]	LEU2	\	Bienert et al. BBA (2014)
**pRS425 *pTPIu* ZmPIP25 H199K**	Zea mais PIP2;5 aquaporin codon-optimized for yeast expression with H199K mutation [PLASMA MEMBRANE]	LEU2	\	Bienert et al. BBA (2014)
**pRS425 *pTPIu* Empty**	TPI promoter, 2μ-plasmid	LEU2	\	Bienert et al. BBA (2014)
