# Supplementary material for: Hyperoxidation of mitochondrial peroxiredoxin limits H2O2‐induced cell death in yeast
Source: EMBO J. 2019 Aug 7;38(18):e101552. doi: 10.15252/embj.2019101552 (PMC6745495; doi:10.15252/embj.2019101552)
Supplement: Supplementary file 5 — Source Data for Figure 4 [file EMBJ-38-e101552-s003.PDF]

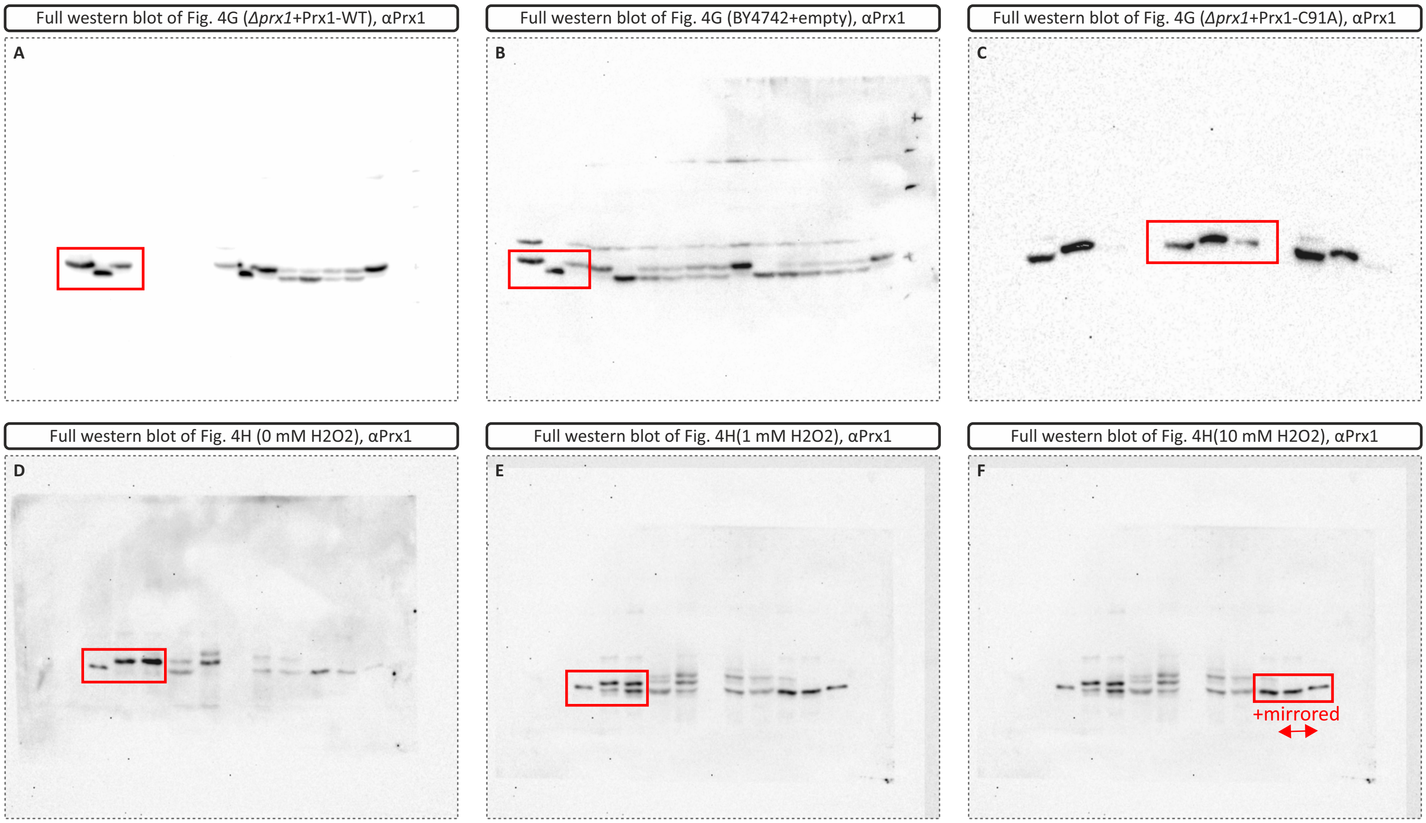

Source Data for Figure 4G,H.

(A-F) Full source raw western blot images for all the cropped versions presented in the paper; the reference to each specific figure is indicated at the top of each blot. Marked with the red rectangle, the relevant area from which the respective presented western blot is cropped.
